# Human Semenogelin 1 Promotes Sperm Survival in the Mouse Female Reproductive Tract

**DOI:** 10.3390/ijms21113961

**Published:** 2020-05-31

**Authors:** Daiki Sakaguchi, Kenji Miyado, Teruaki Iwamoto, Hiroshi Okada, Kaoru Yoshida, Woojin Kang, Miki Suzuki, Manabu Yoshida, Natsuko Kawano

**Affiliations:** 1 Laboratory of Regulatory Biology, Department of Life Sciences, School of Agriculture, Meiji University, Kanagawa 214-8571, Japan; b01133040d@edu.teu.ac.jp; 2 Department of Reproductive Biology, National Research Institute for Child Health and Development, Tokyo 157-8535, Japan; miyado-k@ncchd.go.jp (K.M.); kwjbear@gmail.com (W.K.); sb14536z@st.kitasato-u.ac.jp (M.S.); 3 Division of Male Infertility, Center for Human Reproduction, Sanno Hospital, International University of Health and Welfare, Tokyo 107-0052, Japan; t4iwa@iuhw.ac.jp; 4 Department of Urology, Dokkyo Medical University Saitama Medical Center, Saitama 343-8555, Japan; hirooka@dokkyomed.ac.jp; 5 Faculty of Biomedical Engineering, Toin University of Yokohama, Kanagawa 225-8503, Japan; yoshidak@toin.ac.jp; 6 Misaki Marine Biological Station, School of Science, the University of Tokyo, Kanagawa 238-0225, Japan

**Keywords:** intrauterine sperm survival, IUI, seminal vesicle secretions, human semenogelin 1

## Abstract

Semenogelin 1 (SEMG1), a main component of human seminal plasma, is a multi-functional protein involved in the regulation of sperm motility and fertility. SEMG1 is orthologous to mouse seminal vesicle secretion 2 (SVS2), required for sperm survival in the female reproductive tract after copulation; however, its in vivo function remains unclear. In this study, we addressed this issue by examining the effect of recombinant SEMG1 on intrauterine mouse sperm survival. SEMG1 caused a dose-dependent decrease in mouse sperm motility, similar to its effect on human sperm, but SVS2 had no effect on mouse sperm motility. Mouse epididymal sperm in the presence of 100 µM SEMG1, a concentration that does not affect mouse sperm motility, were injected into the mouse uterus (intrauterine insemination, IUI). IUI combined with SEMG1 significantly increased the survival rate of intrauterine mouse sperm. The effect of SEMG1 on intrauterine sperm survival was comparable with that of SVS2. For clinical applications, three potentially sperm-protecting polypeptides that are easy to handle were designed from SEMG1, but their individual use was unable to mimic the ability of SEMG1. Our results indicate that SEMG1 has potential clinical applications for effective IUI and thereby for safe, simple, and effective internal fertilization.

## 1. Introduction

Mammalian sperm are not initially able to fertilize eggs upon being ejaculated into the female reproductive tract. Sperm acquire their fertilization ability, called capacitation, during their journey through the female reproductive tract [1,2]. This process is accompanied by cholesterol efflux from the sperm plasma membrane [3], tyrosine phosphorylation of axonemal proteins [4,5], and changes in sperm flagellar motility [6]. Capacitated sperm acquire vigorous and asymmetrical flagellar movement with high amplitude. This movement, called hyperactivation, is a critical phenomenon for sperm penetration of the zona pellucida [7]. The capacitated sperm can undergo the acrosome reaction (AR), which is a prerequisite for sperm fusion to eggs.

The seminal plasma has an inhibitory effect on sperm capacitation, and in vitro capacitated sperm reversibly lose their fertilization ability by treatment with seminal plasma [8]. This phenomenon is known as sperm decapacitation and is conserved among mammals, including primates and rodents [9]. Generally, the seminal plasma is a mixture of factors secreted from several accessory organs, including the epididymis, seminal vesicle, and prostate. In particular, seminal vesicle secretion is predicted to play an important role in the regulation of sperm fertility. In fact, the removal of seminal vesicles results in a decline in mouse fertility [10,11,12].

In humans, Semenogelin 1 and 2 (SEMG1 and SEMG2) are major components secreted by the seminal vesicle. These basic fibrous proteins form a coagulum of ejaculated semen [13]. They are cleaved within 20 to 30 minutes after ejaculation by prostate-specific antigen (PSA), a protease released from the prostate, and are converted into a liquid state (liquefaction) [14,15]. Interestingly, one SEMG fragment, a 14 kDa protein isolated from the human seminal plasma termed seminal plasma motility inhibitor (SPMI), inhibits the motility of human sperm [16]. The concentrations of SEMG1 and SEMG2 (SEMGs) as well as SPMI in the liquefied seminal plasma exhibit weak negative correlations with sperm motility [17]. In patients with male infertility, asthenozoospermia, it is possible that the binding of SPMI renders sperm immotile [18]. In this study, we designed two epitope polypeptides, EP1 and EP3, against the SPMI fragment. Both SEMG1 and SEMG2 also include repetitive amino acid units [19]. The rate of molecular evolution of repeats in SEMG2 correlates with levels of female promiscuity in primates [20]. Since the full-length SEMG1 inhibits the protein tyrosine phosphorylation and AR in human spermatozoa, it seems to act as a decapacitation factor, thereby suppressing sperm fertility [21]. Although the mechanism of acceptance of SEMGs has not been fully elucidated, binding of SEMG1 to sperm via adaptor protein EPPIN has been shown [22,23]. Moreover, the SEMG-unbound sperm count is expected to be a relevant parameter for in vivo fertilization based on clinical studies on infertile couples using assisted reproductive technology [24].

In mice, seminal vesicle secretions mainly contain seven proteins, SVS1–7 (SVSs), among which SVS2 is homologous to human SEMG1 [19,25]. We have previously shown that SVS2 acts as a decapacitation factor [25,26]. It binds to the sperm membrane and inhibits sterol efflux accompanying capacitation [25,27]. SVS2 enters the uterus and attaches to the sperm after copulation but is unable to enter the oviduct, at which point the sperm are capacitated [25]. Furthermore, *Svs2*-deficient (*Svs2^−/^^−^*) male mice show striking reductions in fertility due to a spermicidal effect inside the uterine cavity [11]. Thus, SVS2 is indispensable for the intrauterine survival of the sperm by covering the sperm plasma membrane and further layering other components of seminal vesicle secretions (sperm protection). In other words, the detachment of SVS2 from the sperm surface triggers capacitation.

Because SVS2 is crucial for in vivo fertilization in mice, SEMGs may similarly function in sperm protection in humans. Furthermore, SEMGs and/or SVS2 may be useful sperm-protecting agents in other taxa, with implications for increasing the fertilization rate in intrauterine insemination (IUI). To explore potential sperm-protective agents for IUI, we assessed the effects of SEMG1, its polypeptides (EP1 and EP3) [17], fragments of SPMI [16,28], and a repeat sequence in SEMG1 on sperm viability and hyperactivation. Owing to limitations in the use of human gametes, we utilized mouse sperm as a substitute for human sperm.

## 2. Results

### 2.1. Effect of Human SEMG1 on Mouse Sperm

**Figure 1:**
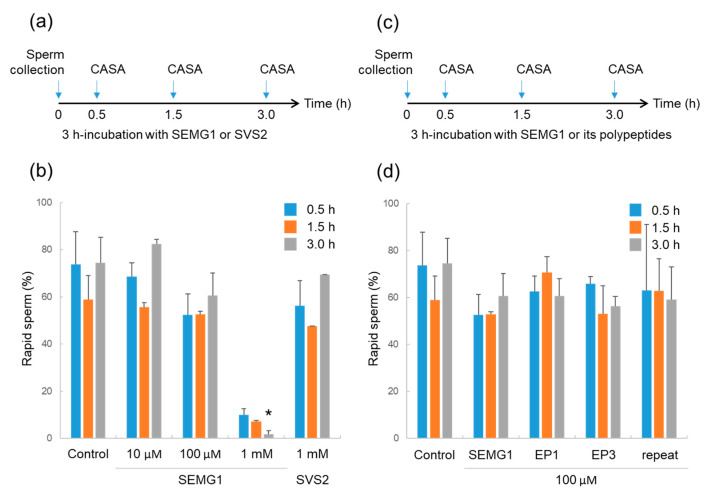
Mouse sperm motility during in vitro incubation for 3 h with human Semenogelin 1 (SEMG1) and its polypeptides. (**a**). Experimental design for determining the appropriate concentration of SEMG1 on epididymal sperm. (**b**). Rates of epididymal sperm displaying rapid motility during 3 h of incubation (*n* = 3). Data are expressed as the average ± standard error of the mean. *p < 0.05, compared with control for the same time. (**c**). Experimental design for testing the effect of human SEMG1 fragmented polypeptides on mouse sperm motility. (**d**). Rates of epididymal sperm displaying rapid motility after 3 h incubation (*n* = 3). Data are expressed as the average ± standard error of the mean.

In order to determine the dose of SEMG1 to be safely tested in vivo, we reconfirmed the positive effect of SEMG1 proteins on sperm motility compared with TYH medium without any additives as a control (Figure 1a). When mouse epididymal sperm were incubated with recombinant 1 mM SEMG1, the percentage of rapid sperm significantly decreased after 3 h incubation (Figure 1b, *p* < 0.05, *n* = 3). In contrast, mouse SVS2 had no effect on sperm motility under the same concentration. Furthermore, 1 mM SEMG1 resulted in lower sperm viability (23.9 ± 2.2%) than untreated sperm (82.7 ± 4.5%). As reported previously [29], 1 mM SEMG1 inhibits sperm motility, but in physiological situations, the inhibitory state is immediately canceled by PSA-mediated digestion of SEMG1. Our results suggest that 1 mM SEMG1 inhibits sperm motility, eventually inducing sperm death after 3 h incubation. Therefore, 100 µM SEMG1, a concentration that does not inhibit sperm motility, was used for a sperm survival assay.

### 2.2. Effects of SEMG1-Derived Polypeptides on Mouse Sperm

**Figure 2:**
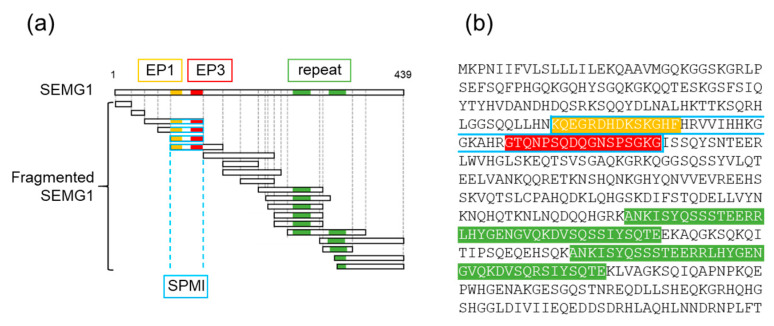
Protein structures of human SEMG1. (**a**) Localization of three polypeptides used in this study. Fragmented SEMG1 is naturally generated in human ejaculated semen by proteolytic activity of prostate-specific antigen (PSA) secreted by the prostate (blue square) [28]. Colors indicate individual polypeptides localized in SEMG1 fragments. (**b**) Amino acid sequences of human SEMG1. Colors indicate individual polypeptides shown in Figure 1c,d.

After SEMG1 is digested with PSA, its fragmented polypeptides exert a physiological effect on sperm fertility [28]. In this study, we focused on three unique polypeptides: EP1 (MW1677.76, pI 9.4), EP3 (MW1658.66, pI 6.3), and Repeat (MW4276.56, pI 6.1) [17,28] (Figure 2a,b). To examine their effects on mouse sperm motility, the epididymal sperm were incubated with each polypeptide at 100 µM for 3 h (Figure 1c). As shown in Figure 1d, all three polypeptides had no effect on sperm motility at every incubation time examined. Therefore, we confirmed the optimum concentration of polypeptides is 100 µM for the sperm survival assay.

### 2.3. Evaluation of Sperm Survival After IUI

**Figure 3:**
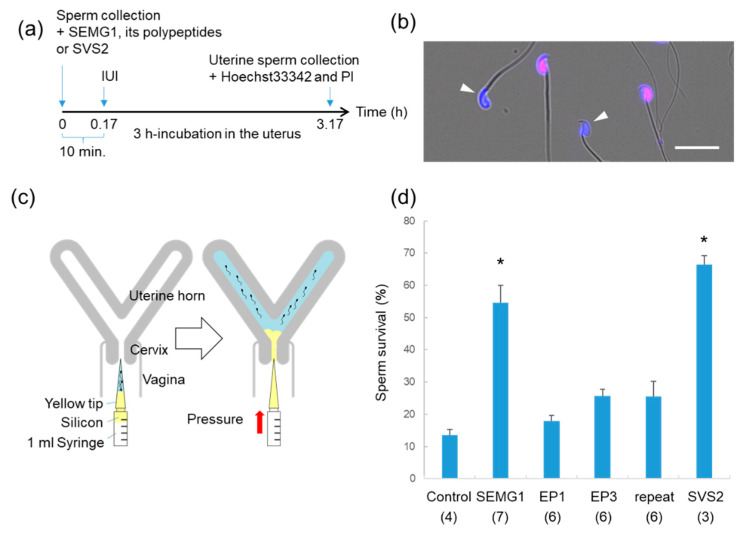
Intrauterine sperm survival after mouse intrauterine insemination (IUI). (**a**) Experimental design for testing intrauterine sperm survival after mouse IUI. (**b**) Image of intrauterine sperm stained with PI and Hoechst33342. White arrows indicate live sperm. Scale bar: 20 µm. (**c**) A schematic diagram of the mouse IUI procedure. Epididymal sperm suspension co-injected with human SEMG1 (100 µM), its fragment polypeptides (100 µM), or mouse seminal vesicle secretion 2 (SVS2) (1 mM) in the uterine cavity. After the sperm injection, silicon was added to the cervix and the uterine cavity in order to prevent a backflow of the sperm suspension. (**d**) Rates of survived sperm in the uterus determined by staining with PI. Parentheses, numbers of female mice examined. Data are expressed as the average ± standard error of the mean. * *p <* 0.05, compared with control sperm.

To explore the protective effect of SEMG1 and its polypeptides on sperm in the female reproductive tract, mouse epididymal sperm mixed with SEMG1 and its polypeptides were injected into the mouse uterus by an IUI technique (Figure 3a). Following sperm injection through the uterine cervix, silicon was added to prevent backflow from the uterus to the vagina (Figure 3c). After 3 h in the uterus, the intrauterine sperm were collected and double-stained with Hoechst33342 and PI (Figure 3b). As determined by PI and Hoechst33342 staining, when epididymal sperm without additives were injected into the uterus, the percentage of live sperm was very low (13.5 ± 1.8%) (Figure 3d). However, the rates of live sperm were significantly higher in the sperm treated with 1 mM mouse SVS2 and 100 µM human SEMG1 (66.3 ± 5.0% and 54.7 ± 13.8%, respectively; *p <* 0.05) than in untreated sperm. These results indicate that human SEMG1 and mouse SVS2 have protective activity on the uterine sperm, and the SEMG polypeptide EP3 tends to increase the sperm survival rate.

### 2.4. Effects of SEMG1 and its Polypeptides on Pre-Incubated Sperm

**Figure 4:**
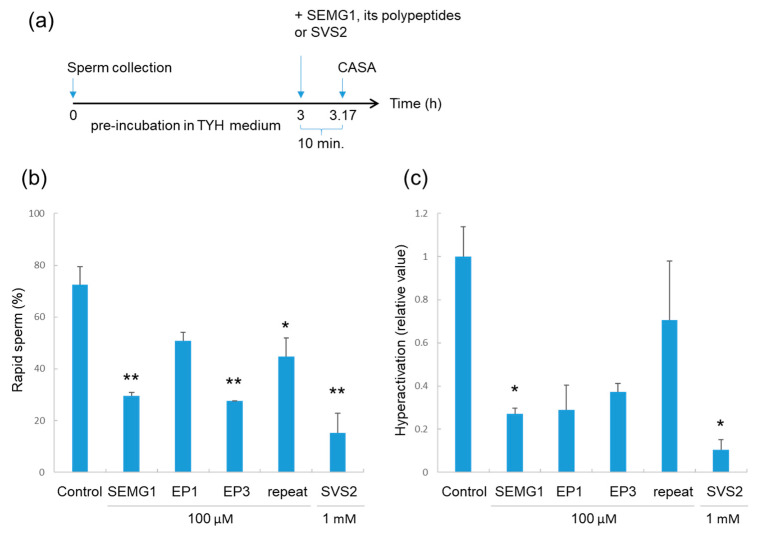
Effect of sperm protective agents on sperm motility after pre-incubation. (**a**) Experimental design for testing short exposure with five factors on sperm motility after 3 h incubation. (**b**) Rates of epididymal sperm displaying rapid motility after 3 h of pre-incubation (*n* = 3). Data are expressed as the average ± standard error of the mean. *p < 0.05 and **p < 0.01, compared with control sperm. (**c**) Rates of epididymal sperm displaying hyperactivated movement after 3 h of pre-incubation (*n* = 3). Data are expressed as the average ± standard error of the mean. * *p <* 0.05, compared with control sperm.

To investigate the relationship between sperm-protection activity and the regulation of sperm motility, we examined the functions of human SEMG1 and its polypeptides on mouse sperm after 3 h of pre-incubation (Figure 4a). When the pre-incubated sperm were exposed to EP1 polypeptide, which lacks sperm-protection activity, for a short period (10 min), motility did not change significantly (*n* = 3) (Figure 4b). However, repeat polypeptide exposure significantly decreased the ratio of rapid spermcompared to the control (*p* < 0.05, *n* = 3) (Figure 4b). Moreover, mouse SVS2, human SEMG1, and its polypeptide EP3 strongly inhibited sperm motility after 3 h of pre-incubation compared with that of untreated sperm (*p* < 0.01, *n* = 3) (Figure 4b). As shown in Figure 1, these factors had no effect on sperm motility, even after a long incubation period (3 h); however, the pre-incubated sperm exhibited reduced motility, even after short-term incubation. These results indicate that the factor conferring a protective effect on sperm in the uterus also inhibits pre-incubated sperm motility.

In order to examine the population of hyperactivated sperm, we re-analyzed the sperm movement after 3 h of pre-incubation with or without human SEMG1 and the EP3 polypeptide on mouse sperm (Figure 4c). When the average hyperactivation rate of control sperm was set at 1.0, human SEMG1 and mouse SVS2 significantly inhibited sperm hyperactivation (*p* < 0.05, *n* = 3). Two SEMG polypeptides, EP1 and EP3, tended to suppress hyperactivation of the sperm.

## 3. Discussion

Human SEMG1 improved intrauterine sperm survival in mice, consistent with the previously reported function of mouse SVS2, which protects against spermicidal attacks in the uterus [11]. Interestingly, human SEMG1 acts as a protectant for mouse sperm beyond species, and its ability corresponds to SVS2, despite amino acid sequence divergence. *Svs2* is a mouse orthologue of human *SEMG1*, but these two genes share partial homology only in the 5′ region; they contain a signal polypeptide and Semg domain in their amino acid sequences [19,25]. Owing to sequence divergence, functional domain(s) in mouse SVS2 and human SEMG1 is unclear. It is possible that SEMG1 interacts with mouse sperm by its positive charge, because both SVS2 and SEMG1 are basic proteins and the sperm targets of SVS2 are sialic acid and GM1 [26]. Therefore, it is likely that SEMG1 also binds to GM1 of mouse sperm and protects against uterine spermicide in this study. Our results have implications for human-assisted reproductive technology, especially IUI, by indicating that SEMG1 and medically-used basic proteins may be useful sperm protectants.

This study showed a linkage between sperm protection in IUI and the short-term inhibition of sperm motility, which provides a basis for screening sperm protection agents; but many questions still remain. Why did human SEMG1 as well as mouse SVS2 have positive effects on sperm survival in the female reproductive tract? Why were SEMG1 and SVS2 negatively related to pre-incubated sperm motility and hyperactivation after a short-time exposure? We speculate that the protective effect on sperm may be related to decapacitation. We have previously shown that mouse SVS2 inhibits sperm capacitation and the acrosome reaction in vitro [25]. In the absence of SVS2, intrauterine sperm exhibit decreased membrane cholesterol levels and ectopic sperm capacitation, resulting in an inability to survive in the female reproductive tract [27]. Moreover, human SEMG1 and its fragments SPMI as well as SVS2, inhibit sperm capacitation [25,30]. Assuming the 3 h pre-incubation in Figure 4 induced sperm capacitation, the effects on sperm protection were correlated with the inhibitory effects on hyperactivation in capacitated sperm. Thus, sperm motility for capacitated spermatozoa may be examined by high-throughput screening methods to identify an effective sperm protectant for IUI.

Among the three SEMG1 polypeptides examined in this study, EP3 is a candidate as a sperm protectant, because the short exposure of EP3 significantly inhibited rapid sperm after the 3 h pre-incubation (Figure 4b) and tended to decrease the population of the hyperactivated sperm (Figure 4c). Since the EP3 polypeptide did not show significant inhibitory effects on the hyperactivation (Figure 4c), the EP3 in the present form may be insufficient for inhibiting sperm capacitation. The EP3, which comprises 17 amino acids (GTQNPSQDQGNSPSGKG), is easily synthesized and handled compared with full-length SEMG1, with a molecular weight exceeding 60 kDa and a high degree of aggregation. Because the intact SEMG1 is difficult to handle for clinical applications as a sperm-protective agent, we need to find functional polypeptides of human SEMG1. In terms of the sperm hyperactivation, EP1 and EP3 likely inhibit the sperm hyperactivation (Figure 4c). Moreover, the EP1 is a basic polypeptide that interacts with SEMG and SVS2. It is possible that the combination of EP1 and EP3, that is SPMI, protects intrauterine sperm survival. Meanwhile, therapeutic proteins have been used, but their effects are frequently blocked by the production of anti-drug antibodies. To lower the risk of anti-drug-antibody production by repeated IUI, the development of an effective sperm-protective polypeptide is a good idea. Although the functional domain of human SEMG1 is still unclear, we will continue to look for functional polypeptides that protect the uterine sperm efficiently.

Mice and humans exhibit substantial differences in properties of reproductive tissues, the reproductive environment, and sexual behavior. In particular, sperm migration across the uterus is still an unexplained phenomenon in human reproduction. In comparison to the time period (a few hours) required for in vivo fertilization in the mouse, it is estimated that human sperm take about 80 h to fertilize in the female reproductive tract [31]. Although our results for mice cannot be directly applied to human reproduction, it is important to evaluate human sperm survival in response to SEMG1 or its fragments in the mouse female reproductive tract. Interestingly, the seminal plasma proteins prevent sperm capacitation and protect intrauterine sperm in both humans and mice, despite differences in the amino acid sequences of SVS2 and SEMG1. Therefore, there are likely conserved systems for sperm protection in mice and humans, and mouse sperm may be a good model for human sperm behavior in vivo. Clarifying the precise functions of SEMG1 in vivo will provide an effective tool for human IUI.

In this study, we provide clear evidence that human SEMG1 protects intrauterine sperm in mice. These results provide a basis for the development of more effective IUI treatments, which are currently recognized as low-probability therapies, requiring safe, effective alternatives.

## 4. Materials and Methods

### 4.1. Animal Care

All animal experiments were performed in an ethical manner after obtaining the approval of the Animal Care Committee of the School of Science, the University of Tokyo (AnimalPlan30-1), and the Meiji University (IACUC15-0014).

### 4.2. Materials

The human seminal vesicle protein SEMG1 was produced by the baculovirus system in *Spodoptera frugiperda* (Sf21) cells [32]. Recombinant SEMG1 proteins were purified using Dynabeads His-Tag Isolation & Pulldown (Invitrogen, Carlsbad, CA, USA). SVS2 proteins were purified from mouse seminal vesicle fluid using a gel filtration column (Bio-Gel P30; Bio-Rad, Tokyo, Japan) as described previously [25]. Fractions containing highly purified SEMG1 and SVS2 proteins were dialyzed, lyophilized, and stored at −80 °C. SEMG1 polypeptides EP1 and EP3 were synthesized by Eurofins Genomics K.K. (Tokyo, Japan), and a repeat polypeptide was synthesized by Scrum Co. (Tokyo, Japan). Each polypeptide was prepared with deionized water and stored at −80 °C until use.

### 4.3. Measurement of Sperm Motility

Epididymal sperm from 3- to 5 month-old Slc:ICR males were dispersed in a 0.1 mL drop of TYH medium (119.37 mM NaCl, 4.78 mM KCl, 1.71 mM CaC1_2_-2H_2_O, 1.19 mM KH_2_PO_4_, 25.07 mM NaHCO_3_, 5.56 mM glucose, 1 mM pyruvic acid, 4 mg/mL BSA, 5 mg/100 mL streptomycin, and 7.5 mg/100 mL penicillin G). After incubation for 10 min at 37 °C in an atmosphere of 5% CO_2_ in air, an aliquot (1.5 × 10^4^ sperm/10 µl) of the sperm suspension was mixed with a 90 µl drop of TYH medium containing seminal vesicle proteins. As depicted in Figure 1a,c, Figure 3a and Figure 4a, sperm suspensions incubated for various durations were transferred into prewarmed counting chambers (20 µm in depth) (Leja Products BV; Nieuw-Vennep, The Netherlands), and sperm movement was captured using a video camera (acA780-75gm) (Basler AG, Ahrensburg, Germany) mounted on a phase-contrast microscope (BX51) (Olympus, Tokyo, Japan) at 37 °C. Sperm motility parameters were quantified by computer-assisted semen analysis (CASA) using the Sperm Class Analyzer (SCA) system (Microptic; Barcelona, Spain). At least 200 sperm were examined for each sample. Rapid sperm were defined as sperm whose curvilinear velocities were over 120 µm/s, following the manufacturer’s instruction of SCA.

Hyperactivated sperm were calculated from the CASA parameters in accordance with the criteria of Goodson, whose algorithms were established by using Support Vector Machines (SVM) [33]. Briefly, we calculated the two SVM parameters from the CASA parameters:

SVM1; (0.0388 × VAP) + (0.0335 × VSL) + (0.0225 × VCL) − (0.0248 × ALH) + (0.0051 × BCF) − 10.9540

SVM2; (0.0123 × VAP) − (0.1034 × VSL) + (0.0307 × VCL) + (0.0427 × ALH) + (0.0175 × BCF) − 3.6222

If both SVM1 and SVM2 were positive values, we designated the sperm as hyperactivated. The relative value of hyperactivated sperm was calculated by comparing it to the average of the hyperactivation rate of the control sperm from the same specimen.

### 4.4. Sperm Survival Assay after IUI

To evaluate sperm survival in females, sperm were isolated from the epididymis of 3- to 5-month-old ICR male mice and directly injected into the uterus of 2-month-old ICR female mice with a syringe. Before sperm collection, the female mice were determined to be in the estrus phase by vaginal smear cytology [34]. As depicted in Figure 3a, a 50 µl aliquot of the epididymal sperm suspension (5 × 10^7^ sperm/mL) was prepared in a yellow tip attached to a 1 mL syringe containing silicon (Beckman Coulter, Brea, CA, USA) and then injected from the cervix into the uterus with or without seminal vesicle proteins. Finally, 20 to 50 µl silicon entered the cervix and the uterine body and was used as a substitute for a copulatory plug. The stored seminal vesicle proteins and polypeptides were dissolved in deionized water, diluted twice in 2× concentrated TYH medium, and mixed with an approximately one-tenth volume of the sperm suspension. At 3 h after sperm injection, female reproductive tracts from the cervix to oviduct were excised following cervical dislocation. The intrauterine sperm were collected by MµltiFlex Round Tips (Sorenson BioScience, Inc., Salt Lake City, UT, USA) and stained with 5 µg/mL Hoechst33342 (Thermo Fisher Scientific, Waltham, MA, USA) and 10 µg/mL propidium iodide (PI; Sigma-Aldrich, St. Louis, MO, USA). Within 15 min after sperm collection, sperm fluorescence stained with PI and Hoechst33342 was observed and captured using a BZ-X700 fluorescent microscope (Keyence, Tokyo, Japan). Double staining with PI and Hoechst33342 indicated dead sperm, and PI-negative but Hoechst33342-positive findings indicated live sperm. The sperm survival rate was calculated by dividing the number of PI-negative sperm by the number of Hoechst33342-positive sperm (≥200 sperm examined).

### 4.5. Statistical Analysis

Data are presented as mean values ± standard error of the mean (*n* ≥ 3), unless otherwise stated. To compare against control in each experiment, results were analyzed using one-way-ANOVA with Tukey-Kramer post hoc tests, with a significance threshold of *p* < 0.05.

## 5. Conclusions

Human SEMG1 protects the sperm survival in mouse reproductive tract. The effect of SEMG1 on intrauterine sperm survival was comparable with that of mouse SVS2. For clinical applications, three potentially sperm-protecting polypeptides that are easy to handle were designed from SEMG1, and further investigations about polypeptides of SEMG1 will open new horizons in the human effective IUI.
